# Musical rhythm production associates with cognitive functioning in healthy older adults

**DOI:** 10.1002/alz70858_107637

**Published:** 2025-12-25

**Authors:** Aaron Colverson, John B. Williamson, Ronald A. Cohen, Katherine P Rankin, Stephanie Barsoum, Xavier A. Velez, Samantha Alicea, Romina Valerio, Mariana Occhiuzzi, Ashley Wu, Isabel Elaine Allen

**Affiliations:** ^1^ University of California, San Francisco, CA, USA; ^2^ University of Florida, Gainesville, FL, USA; ^3^ Memory and Aging Center, Weill Institute for Neurosciences, University of California San Francisco, San Francisco, CA, USA; ^4^ Global Brain Health Institute (GBHI), University of California San Francisco, San Francisco, CA, USA; ^5^ Multiscale Imaging and Integrative Biophysics Unit, National Institute on Aging, NIH, Baltimore, MD, USA; ^6^ Global Brain Health Institute, University of California, San Francisco, CA, USA, San Francisco, CA, USA, San Francisco, CA, USA; ^7^ Memory and Aging Center, UCSF Weill Institute forNeurosciences, University of California, San Francisco, San Francisco, CA, USA, San Francisco, CA, USA

## Abstract

**Background:**

Music‐based interventions associate with improvements in cognitive and social outcomes in older adults living with and without dementia. Understanding age‐related alterations of rhythm performance in older adults is necessary to understand how neural mechanisms change in the context of Alzheimer's disease and related dementias (ADRDs). However, relatively little is known about ways in which healthy aging changes how we process musical rhythm. Therefore, we designed a rhythmic musical activity to understand relationships between aging‐related changes in cognitive functioning and rhythm production. We hypothesized that healthy older adults would perform worse on more complex musical rhythms compared with younger adults and that this difference would correlate with cognitive processes affected by aging.

**Method:**

Fifty‐four healthy participants (ages 18‐35: *n* = 29; ages 55‐89: *n* = 25) learned and performed 30 musical rhythm exercises of increasing complexity over three learning trials and one performance trial. Complexity of rhythm exercises was determined by relative amount of syncopation and number of beats in a given rhythmic phrase. Participants also underwent cognitive testing that included measures of processing speed, working memory, and set shifting. Mixed effects general linear models determined the associations between cognitive performance and rhythm production.

**Result:**

Age group (young or old) significantly affected the learning of musical rhythms (t = ‐7.02, *d* = ‐1.94, *p* <0.001), with older adults learning more slowly than younger. Additionally, controlling for age and education, participants’ musical rhythm performance accuracy was predicted by their scores on processing speed, working memory and set shifting tests. The effect of age was stronger on rhythm performance than on our cognitive metrics. This result suggests independent impacts of aging on rhythm relative to changes in working memory, processing speed, and set shifting.

**Conclusion:**

This study found that healthy aging negatively impacts musical rhythm production, beyond age‐related changes to processing speed, working memory, and other executive functions. Non‐pharmacological interventions remain desperately needed for ADRDs, and music‐based interventions are increasingly being used. Thus, more neuroscientifically‐grounded investigations are needed to reveal underlying cognitive mechanisms and clarify why engaging in musical learning and performance can positively impact the brain health of adults experiencing aging and dementia.

